# The relationship between Insulin‐like Growth Factor 1, sex steroids and timing of the pubertal growth spurt

**DOI:** 10.1111/cen.12682

**Published:** 2015-01-08

**Authors:** T.J. Cole, M.L. Ahmed, M.A. Preece, P. Hindmarsh, D.B. Dunger

**Affiliations:** ^1^ Population Policy and Practice ProgrammeUCL Institute of Child HealthLondonUK; ^2^ Department of PaediatricsChildren's HospitalOxfordUK; ^3^ Genetics and Genomic Medicine ProgrammeUCL Institute of Child HealthLondonUK; ^4^ Developmental Endocrinology Research GroupUCL Institute of Child HealthLondonUK; ^5^ Department of PaediatricsUniversity of Cambridge School of Clinical MedicineCambridgeUK

## Abstract

**Objective:**

Progress through puberty involves a complex hormonal cascade, but the individual contributions of hormones, particularly IGF‐1, are unknown. We reanalysed Chard growth study data to explore the tempo of puberty based on changes in both height and hormone levels, using a novel method of growth curve analysis.

**Design and Subjects:**

Schoolboys (*n* = 54) and girls (*n* = 70) from Chard, Somerset, England, recruited in 1981 at age 8/9 and followed to age 16.

**Measurements:**

Every 6 months, height and Tanner stages (genitalia, breast, pubic hair) were recorded, and in a subsample (24 boys, 27 girls), blood samples were taken. Serum IGF‐1, testosterone (boys) and oestradiol (girls) were measured by radioimmunoassay. Individual growth curves for each outcome were analysed using variants of the super‐imposition by translation and rotation (SITAR) method, which estimates a mean curve and subject‐specific random effects corresponding to size, and age and magnitude of peak velocity.

**Results:**

The SITAR models fitted the data well, explaining 99%, 65%, 86% and 47% of variance for height, IGF‐1, testosterone and oestradiol, respectively, and 69–88% for the Tanner stages. During puberty, the variables all increased steeply in value in individuals, the ages at peak velocity for the different variables being highly correlated, particularly for IGF‐1 *vs* height (*r* = 0·74 for girls, 0·92 for boys).

**Conclusions:**

IGF‐1, like height, the sex steroids and Tanner stages, rises steeply in individuals during puberty, with the timings of the rises tightly synchronized within individuals. This suggests that IGF‐1 may play an important role in determining the timing of puberty.

## Introduction

The period of rapid growth in height that occurs at adolescence is unique to humans. No other primate species, including the chimpanzee, has such a dramatic increase in height velocity although the male chimpanzee does undergo a period of rapid gain in muscle mass.^1^ The adolescent growth spurt begins on average 2 years earlier in girls than boys, but within each gender there is enormous variation in the timing of pubertal development. It is generally accepted that the peak in height velocity at adolescence is driven by a cascade of hormones, primarily initiated by a rise in sex steroid and followed by increases in growth hormone and the related insulin‐like growth factor 1 (IGF‐1).^2^, ^3^ However, deciphering the individual contributions of these hormones, particularly IGF‐1, to the timing and intensity of pubertal growth acceleration has proved difficult.

It has been reported that in individuals IGF‐1 peaks at Tanner stage 5,^4^ that raised IGF‐1 is associated with early puberty^5^, ^6^ and that IGF‐1 measured cross‐sectionally increases both with age and pubertal stage through puberty, peaking in girls at Tanner stage 4.^5^, ^7^ However, there is uncertainty about the longitudinal pattern of change in IGF‐1 in individuals, and how it might relate to the timing of the rise in sex steroids (testosterone in boys and oestradiol in girls) or the age at peak height velocity (PHV).

The aim of this study was to use a novel method for the analysis of growth curves which permits derivation of mean growth curves allowing for variation in individual timing and intensity of puberty, to examine the relationship with parallel changes in the principal hormones IGF‐1, testosterone and oestradiol, and the Tanner stages. The study took advantage of the detailed longitudinal data collected by the Chard growth study in the 1980s.

## Methods

The Chard 2 growth study^4^ was a longitudinal study of 54 boys and 70 girls recruited in 1981 from four primary schools in the town of Chard, Somerset, England. Parents were written to when the children were aged 8–9 years, in the penultimate year before transfer to the secondary school. The letter explained that measurements of height and weight would be taken together with puberty ratings and a blood sample. Measurements were taken every 6 months using appropriate equipment brought from London and calibrated before each measuring session. The measurements and puberty ratings (Tanner stages for genitalia, breast and pubic hair)^8^, ^9^ were done by two experienced auxology technicians from the growth clinic at Great Ormond Street Hospital in London, each with a regularly assessed technical error of height measurement close to 0·2 cm. The girls were seen by a female measurer and the boys by a male. Bloods were taken from a subsample of children (24 boys, 27 girls) whose parents had given permission, by a medical doctor experienced at taking blood from children. Concentrations of serum IGF‐1 and testosterone (boys) were measured by radioimmunoassay,^10^, ^11^ while serum oestradiol (girls) was measured with the double antibody Diagnostics Products Corp kit (Llanberis, Wales, UK). Interassay precision was 12%, <10% and <10% for the three assays. The study had research ethics approval from the Joint Research Ethics Committee of the Institute of Child Health and Great Ormond Street Hospital.

The longitudinal data for individuals plotted against age can be viewed as ‘growth’ curves, which is a novel approach for the blood‐based measures and Tanner stages. The super‐imposition by translation and rotation (SITAR) method^12^ was used to summarize these growth curves, estimating for each individual their magnitude of peak velocity and the age when it occurred. SITAR has previously been used to model height growth in puberty,^13^ and the effect on height growth of oxandrolone in Turner Syndrome^14^ and calcium supplementation in Gambian adolescents.^15^ It has also been used to model weight gain in infancy.^16^, ^17^, ^18^, ^19^, ^20^

In SITAR, the individual fitted curves are assumed to be the same underlying shape as the mean curve, but individual curves are allowed to differ from the mean curve in three respects, called *size*,* tempo* and *velocity*, which adjust the individual curves to match the mean curve. The *size* parameter shifts each individual's curve up or down, so it reflects their size relative to the average. The *tempo* parameter shifts each individual's curve left or right and reflects their timing of puberty or age at peak velocity. The *velocity* parameter stretches or shrinks the age scale for each individual, reflecting their rate of passage through puberty; this has the effect of making the growth curve steeper or shallower and hence increasing or decreasing peak velocity. In this way, each individual's velocity curve is transformed to match the mean velocity curve, at the same time estimating individual puberty timing and intensity. The values of size, tempo and velocity are estimated for each individual as random effects, along with the estimate of the mean curve as a cubic regression spline.

The term *tempo* was used by Tanner in two different ways, the age at peak velocity and the rate of maturation,^21^ each being effectively the inverse of the other (the earlier the age at peak velocity, the faster the rate of maturation). Here, tempo is used explicitly to mean the relative age at peak velocity, that is the timing of puberty, while a separate term, velocity, is used to describe the rate of maturation or intensity of puberty, which manifests itself as the relative peak velocity.

The fitted models used regression splines with 3 degrees of freedom for the hormones and 5 degrees of freedom for height, and the hormones were power‐transformed before fitting (square root for IGF‐1 and log for testosterone and oestradiol), and back‐transformed for display. Age at peak velocity was obtained as the age when the first derivative of the back‐transformed spline curve was maximal; its standard error was obtained with the bootstrap.

The Tanner stage data were analysed using a modified SITAR method that fits a logistic mean curve and adjusts for tempo (timing) and velocity (intensity), but not size.^13^ The size adjustment is not needed as all individuals progress through the same five stages. The slope of the logistic curve is steepest at the midway point, so for Tanner stage, ‘peak velocity’ is in stage 3 and the tempo parameter corresponds to the mean age in stage 3.^13^ Models were fitted using the nlme and sitar libraries in R.^22^, ^23^

## Results

Table 1 gives the numbers of subjects and measurements. SITAR models were fitted to height and IGF‐1 in both sexes, and to testosterone in boys and oestradiol in girls.

**Table 1:** Summary statistics for age at peak velocity (years) by sex

Measure	Boys			Girls		
	Mean (SE)	SD	*N*/*n* a	Mean (SE)	SD	*N*/*n* a
Height	13·9 (0·04)	0·97	54/510	12·3 (0·04)	1·03	70/733
IGF‐1	13·2 (0·1)	0·93	24/320	12·0 (0·1)	0·76	27/361
Testosterone	13·5 (0·1)	1·04	24/321	–	–	–
Oestradiol	–	–	–	12·7 (0·8)	0·77	27/326
Tanner genital stage	13·1 (0·2)	1·01	54/511	–	–	–
Tanner breast stage	–	–	–	12·3 (0·2)	1·27	70/738
Tanner pubic hair stage	13·5 (0·3)	0·96	54/507	12·8 (0·4)	1·13	70/738

a *N* = no. subjects, *n* = no. measurements.

**Figure 1:**
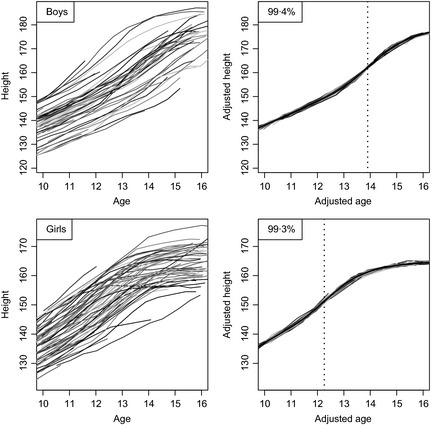
Growth curves of height in boys (above, *n* = 54) and girls (below, *n* = 70), unadjusted (left) and after super‐imposition by translation and rotation (SITAR) adjustment (right). The mean curves are shown in black, and the ages at peak height velocity are shown as dotted lines.

Fig. 1 illustrates the SITAR method applied to height. The left panels show the individual height curves, for boys (top left) and girls (bottom left), colour‐coded with eight colours. Fitting mean curves to the data, treating them as cross‐sectional, gives residual SDs of 7·4 cm in boys and 5·8 cm in girls. The right panels show the same growth curves after SITAR adjustment, and the effect of the adjustment is to superimpose the individual curves; the residual SDs about the fitted curves are 0·6 cm in boys (top right) and 0·5 cm in girls (bottom right), <10% of the unadjusted residual SDs. So the variance is <1% of the unadjusted variance, and the SITAR adjustment explains more than 99% of the variance (the values shown top left in each panel). These residual SDs are similar to those resulting from fitting individual Preece–Baines curves to the data,^24^, ^25^ showing that the SITAR adjustment has accounted for effectively all the underlying interindividual growth variability.

The mean curves are shown in the right panels in black (largely obscuring the individual curves, as they are so close together), and the mean ages at PHV are shown with vertical dotted lines: 13·9 years for boys and 12·3 years for girls. The corresponding mean peak velocities are 9·9 cm/year for boys and 8·0 cm/year for girls.

**Figure 2:**
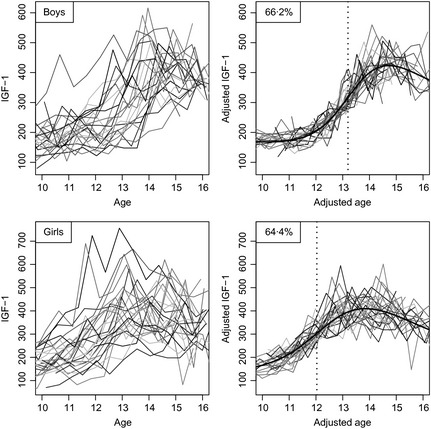
Growth curves of IGF‐1 in boys (above, *n* = 24) and girls (below, *n* = 27), unadjusted (left) and after super‐imposition by translation and rotation (SITAR) adjustment (right). The mean curves are shown in black, and the ages at peak velocity are shown as dotted lines.

Fig. 2 illustrates the SITAR analysis for IGF‐1 in the two sexes. The individual curves (left: boys top, girls bottom) are much noisier than for height, and the underlying curve shape is not obvious. However, the SITAR adjustment is effective at superimposing the curves (right), and it explains two‐thirds of the variance. The mean curves (in black) rise steeply, the most rapid rise occurring at 13·2 and 12·0 years by sex. The curves themselves subsequently peak and fall in both sexes, the peak IGF‐1 levels occurring at 14·5 years in boys and 13·8 years in girls.

The correlations between the IGF‐1 SITAR size and velocity parameters in individuals are highly significant (0·8 in boys and 0·6 in girls), so subjects whose mean IGF‐1 is high tend to progress more rapidly through puberty. Conversely, the correlations between IGF‐1 size and tempo are small and insignificant (<0·1), indicating that the age of puberty onset is unrelated to mean IGF‐1. However tempo and IGF‐1 at age 10 are significantly negatively correlated (r = −0.4 in both sexes, p = 0.02), so that a higher IGF‐1 at this age implies earlier puberty.

**Figure 3:**
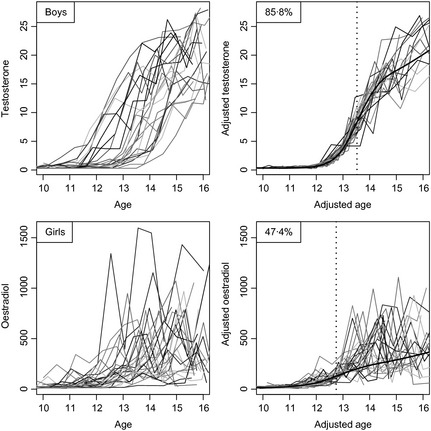
Growth curves of testosterone in boys (above, *n* = 24) and oestradiol in girls (below, *n* = 27), unadjusted (left) and after super‐imposition by translation and rotation (SITAR) adjustment (right). The mean curves are shown in black, and the ages at peak velocity are shown as dotted lines.

Fig. 3 shows the sex steroids testosterone in boys (top) and oestradiol in girls (bottom), in the same format as Figs 1 and 2. The unadjusted curves are even more noisy than for IGF‐1, but despite this the SITAR adjustment explains 86% of the variance in testosterone and 47% in oestradiol. Even after adjustment oestradiol is very noisy past peak velocity. Both mean curves show a steep rise, but unlike IGF‐1 they continue to rise into adulthood. Ages at peak velocity are 13·5 and 12·7 years for testosterone and oestradiol, respectively.

The modified SITAR models used for Tanner stage also fitted the data well (genitalia 69% of variance explained, breast 88%, boys pubic hair 74%, girls pubic hair 79%). The process of adjustment is well illustrated in an earlier publication.^13^

Table 1 summarizes the mean ages at peak velocity for the measurements by sex, with their standard errors which (except for oestradiol) are small. Peak velocity occurs consistently earlier in girls than boys: by 1·6 years for height, 1·2 years for IGF‐1 and 0·7–0·8 years for oestradiol *vs* testosterone and the Tanner stages. In addition, in both sexes, peak velocity occurs earlier for IGF‐1 than for height and the sex steroids.

The standard deviations (SDs) of the individual ages at peak velocity are also shown in Table 1; they are obtained as the SDs of the tempo random effects for each measurement. The individual growth curves can be thought of as the mean curve shifted left or right by an amount corresponding to the individual's tempo random effect, that is the difference between their own age at peak velocity and the mean age at peak velocity; a negative tempo effect here indicates early puberty and a positive value indicates late. These SDs, which indicate the variability of age at puberty onset, range from 9 to 15 months in girls and 11 to 12 months in boys.

**Figure 4:**
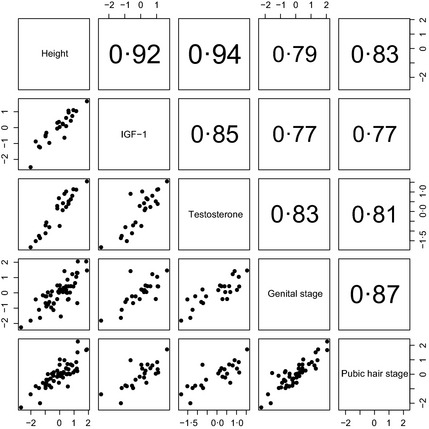
Scatterplot of tempo random effects for height, IGF‐1, testosterone and Tanner genital and pubic hair stage in boys. The correlations (above the diagonal) are all highly significant (*P* < 0·0001).

**Figure 5:**
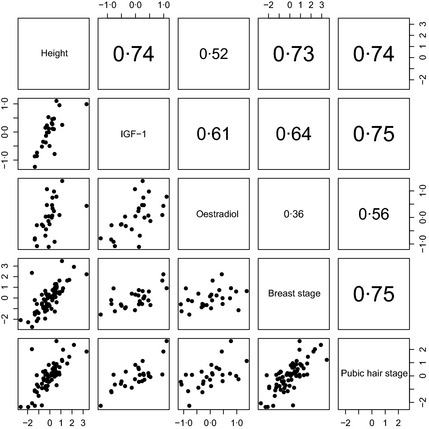
Scatterplot of tempo random effects for height, IGF‐1, oestradiol and Tanner breast and pubic hair stage in girls. The correlations (above the diagonal) are all significant (*P* < 0·01) bar one.

It is reasonable to expect each individual to have the same underlying pubertal tempo according to all five measures. This means that their tempo random effects by the different measures ought to be correlated – they should be ranked similarly according to all five measures. Fig. 4 (boys) and Fig. 5 (girls) examine this by showing the correlation matrix and scatterplot matrix for the tempo random effects based on height, IGF‐1, the sex steroids and the Tanner stages. To read off the values for a given pair of variables, look at the rows and columns containing the two variable names. There are two cells where the rows and columns intersect – the one above the diagonal contains the correlation and the one below contains the corresponding scatterplot.

The correlations are indeed nearly all large and significant and are appreciably larger for boys than girls. The largest correlations, for boys height *vs* IGF‐1 and testosterone (*r* = 0·92 and 0·94, respectively, *P* < 0·0001) are remarkably high, while for girls the oestradiol correlations with height and IGF‐1 are lower (*r* = 0·52 and 0·61) although still highly significant (*P* < 0·005). Only for oestradiol *vs* breast stage is the correlation <0·5 (although still close to significance, *P* = 0·07). The scatterplots in Figs 4 and 5 confirm the linearity of the associations between random effects.

## Discussion

The results show the success of SITAR in modelling the complexities of the adolescent growth spurt. The growth pattern for height is well known (Fig. 1), and the SITAR model fitted to this and similar data sets consistently explains more than 99% of the variance.^12^, ^13^, ^14^, ^15^ In this cohort, mean age at PHV was 12·3 years in girls and 13·9 years in boys, matching the original report from the Chard study,^4^ with mean peak velocity 8·0 cm/year in girls and 9·9 cm/year in boys, somewhat smaller than reported originally (8·8 and 10·9)^4^ or by Tanner (9·0 and 10·3).^26^

Super‐imposition by translation and rotation was also effective in modelling the complex changes in IGF‐1. It was constant during childhood but rose sharply after age 10 in girls and 11 in boys, with peak velocity occurring 2 years later and peak level 2 years after that. A similar pattern was shown by Sorensen,^5^ who plotted mean IGF‐1 in girls against Tanner breast stage, where it peaked at stage 4. The age at peak velocity for IGF‐1 was remarkably highly correlated with that for height. IGF‐1 levels at age 8 years have been shown to be predictive of age at menarche in a recent longitudinal study,^6^ and the role of IGF‐1 in the initiation and progression through puberty has recently been debated.^3^

Testosterone and oestradiol also rose steeply in puberty, and continued to rise afterwards. The timing of their rise was strongly correlated with that for IGF‐1 and height. SITAR adjustment for testosterone and oestradiol explained 86% and 47% of the variance, the poorer fit for oestradiol and its lower correlation with other markers probably reflecting variability due to the menstrual cycle in postmenarcheal girls (whose timing was not recorded), plus the fact that the blood samples were taken in daytime rather than at night.^27^

The age at peak velocity for the Tanner stages (effectively the age in stage 3 due to the assumed logistic model) was similarly closely related to the timings of the other markers (see Figs 4 and 5), despite the inherent noise in the measurements.

Can we decipher the relative roles of these hormones in the initiation and progression of the adolescent growth spurt? It is generally accepted that the initiation of puberty relates to hypothalamic pulsatile release of GnRH and in particular pituitary secretion of predominantly LH over FSH.^28^ These changes lead to gonadal maturation and critical changes in oestradiol (directly from the ovary in girls or indirectly by aromatization of testosterone in boys) which prime pituitary GH release and increases in circulating IGF‐1. In rats, there is evidence that IGF‐1 affects the timing of puberty in females via its effects on LHRH^29^ and KiSS‐1,^30^ although the patterns of growth hormone secretion and IGF‐1 concentration in rats differ from those in humans, not least because they are sexually dimorphic.

The impressive tempo‐related SITAR correlations conform with this projected sequence, although the rise in IGF‐1 precedes that for the sex steroids in individuals. This may simply reflect the fact that in early puberty, sex steroid concentrations are higher at night than in the day.^27^ The Chard subjects were sampled during the day, so this would underestimate concentrations in the girls up to breast stage 3 and the boys with testicular volume <10 ml.

Do the data provide any insights into the relationships between higher IGF‐1 in childhood and advanced tempo? IGF‐1 levels in childhood are associated with the rate of passage through puberty, and IGF‐1 at age 10 is inversely associated with the timing of puberty. So the findings support the idea that a higher prepubertal IGF‐1 predisposes to earlier puberty. Similarly, the reported association of the timing of puberty with IGF‐1 levels 50 years later^31^ squares with the findings here.

The factors that indicate GnRH pulsatility and timing of puberty are still unknown, although new players such as kisspeptin have been identified.^2^ The timing of pubertal onset is clearly partly genetic^32^ but early developmental changes and rates of weight gain are also critical,^33^, ^34^ which may explain associations between IGF‐1 and tempo of pubertal growth.^35^ Whether IGF‐1 levels have a role in the initiation of pubertal development, as has been suggested,^36^ remains unproven.

The data also reflect the biological discordance between the roles of IGF‐1 and sex steroids in pubertal development. The rises in GH and IGF‐1 are transient, and after reaching a peak their concentrations decline into adulthood despite ongoing exposure to sex steroids in concentrations known to induce GH gene transcription. This might imply a resetting of the hypothalamic GH regulatory peptides, GH releasing hormone and somatostatin. In contrast oestradiol/testosterone concentrations continue to rise into adulthood, reflecting their role in achieving full sexual development and reproductive capacity.

The SITAR growth curve analysis has shown its ability to detect individual growth curves even among noisy hormone data and has demonstrated for the first time that ages at peak velocity for the different measures are highly correlated. With more recent hormone assays and larger sample sizes, it should provide further evidence as to the endocrinological patterns that underlie pubertal onset and progression.

So in conclusion, the findings demonstrate that individuals experience a sharp pubertal rise in IGF‐1, and the age at peak IGF‐1 velocity is closely linked to the corresponding ages at peak velocity in sex steroids, height and Tanner stages. This suggests that IGF‐1, as well as the other variables, may play an important role in the timing of puberty.

## Disclosure statement

The authors have nothing to disclose.

## Funding

TJC is funded by UK Medical Research Council grant MR/J004839/1.
